# Combining Genome Surveillance and Metadata To Characterize the Diversity of Staphylococcus aureus Circulating in an Italian Hospital over a 9-Year Period

**DOI:** 10.1128/spectrum.01010-23

**Published:** 2023-07-17

**Authors:** U. Postiglione, G. Batisti Biffignandi, M. Corbella, C. Merla, E. Olivieri, G. Petazzoni, E. J. Feil, C. Bandi, P. Cambieri, S. Gaiarsa, M. Brilli, D. Sassera

**Affiliations:** a Department of Biology and Biotechnology, University of Pavia, Pavia, Italy; b Microbiology and Virology Unit, Fondazione IRCCS Policlinico San Matteo, Pavia, Italy; c Istituto Zooproflattico Sperimentale della Lombardia e dell’Emilia Romagna, Pavia, Italy; d The Milner Centre for Evolution, Department of Biology and Biochemistry, University of Bath, Bath, United Kingdom; e Department of Bioscience, University of Milan, Milan, Italy; f Fondazione IRCCS Policlinico San Matteo, Pavia, Italy; University of Arkansas for Medical Sciences

**Keywords:** *Staphylococcus aureus*, antibiotic resistance, community acquired, genomic surveillance, hospital acquired, risk factors

## Abstract

Staphylococcus aureus is an opportunistic pathogen and a leading cause of morbidity and mortality worldwide. Genomic-based surveillance has greatly improved our ability to track the emergence and spread of high-risk clones, but the full potential of genomic data is only reached when used in conjunction with detailed metadata. Here, we demonstrate the utility of an integrated approach by leveraging a curated collection of clinical and epidemiological metadata of S. aureus in the San Matteo Hospital (Italy) through a semisupervised clustering strategy. We sequenced 226 sepsis S. aureus samples, recovered over a period of 9 years. By using existing antibiotic profiling data, we selected strains that capture the full diversity of the population. Genome analysis revealed 49 sequence types, 16 of which are novel. Comparative genomic analyses of hospital- and community-acquired infection ruled out the existence of genomic features differentiating them, while evolutionary analyses of genes and traits of interest highlighted different dynamics of acquisition and loss between antibiotic resistance and virulence genes. Finally, highly resistant clones belonging to clonal complexes (CC) 8 and 22 were found to be responsible for abundant infections and deaths, while the highly virulent CC30 was responsible for rare but deadly episodes of infections.

**IMPORTANCE** Genome sequencing is an important tool in clinical microbiology, as it allows in-depth characterization of isolates of interest and can propel genome-based surveillance studies. Such studies can benefit from *ad hoc* methods of sample selection to capture the genomic diversity present in a data set. Here, we present an approach based on clustering of antibiotic resistance profiles that allows optimal sample selection for bacterial genomic surveillance. We apply the method to a 9-year collection of Staphylococcus aureus from a large hospital in northern Italy. Our method allows us to sequence the genomes of a large variety of strains of this important pathogen, which we then leverage to characterize the epidemiology in the hospital and to perform evolutionary analyses on genes and traits of interest. These analyses highlight different dynamics of acquisition and loss between antibiotic resistance and virulence genes.

## INTRODUCTION

Staphylococcus aureus is a globally disseminated bacterial species capable of thriving both free-living in the environment and associated with eukaryotic hosts (1). In humans, S. aureus is present in the nasal microbiome of about 30% of the healthy population (2), where it usually is a harmless commensal; however, it can also act as an opportunistic pathogen on immune-deficient hosts or body sites exposed during surgery. S. aureus can cause a plethora of clinical manifestations, ranging from superficial skin infections to invasive, difficult to eradicate, and potentially fatal conditions, such as bacteremia, pneumonia, endocarditis, and osteomyelitis (3). A key factor proposed to explain the success of S. aureus both as a colonizer and as a pathogen is the ability to acquire novel genes and elements (e.g., transposons, bacteriophages, insertion sequences, pathogenicity islands) by horizontal gene transfer (HGT), although chromosomal mutations in the core genome can also play a key role (4). The frequent gain (or loss) of pathogenicity and resistance genes underpins the rapid evolution of high-risk clones, such as those resistant to methicillin (methicillin-resistant S. aureus [MRSA]) (5) that are now widespread in both health care and community settings.

A central question of S. aureus epidemiology concerns the extent of overlap between resistant clones circulating in health care settings, which are frequently exposed to antibiotics, and those in the community, which are broadly considered to be more susceptible to antibiotics (6). Early studies that compared community- and healthcare-acquired (CA and HA) strains highlighted key molecular differences, such as differential expression of the cytotoxin Panton-Valentine leukocidin (PVL, more common in CA strains) (7), different staphylococcal chromosomal cassette (SCC) *mec* types (types IV and V are more common in, but not restricted to, CA strains) (6–8). In addition to these genotypic characteristics, it was shown that CA strains tend to affect healthier and younger patients, mostly causing minor skin and soft tissue infections (9). With the advent of whole-genome sequencing (WGS), more exhaustive comparisons have been performed which challenged the view that CA and HA strains can be clearly distinguished on the basis of genomic features (10–16).

S. aureus was the first species for which population-scale genome sequencing was exploited to understand local and global epidemiology of high-risk clones (17). Subsequent studies helped to determine which clones were more frequent in specific settings and worldwide (18–20) and in environments (21, 22). These population-wide studies provide important baseline data and complement studies focusing on specific lineages at global or local levels (23, 24). These data have shed light on broad aspects of S. aureus epidemiology, such as hypervirulence, specific lineages, and the reconstruction of transmission chains during outbreaks (25–27).

Studies based on sequencing representative samples of the S. aureus population “‘snapshots”’ (20) and studies focused on individual lineages are therefore both valuable for understanding how the frequency of specific clones may change over time and from region to region. However, these approaches may not capture the full diversity of the population, and rare genotypes may be overlooked. Here, we take an alternative approach, aimed to maximize the diversity of strains for sequencing in hospital settings by exploiting archived antibiograms as proxies of genetic relatedness in a semisupervised sampling. We apply our strategy on a 9-year data set from an Italian hospital, and we show that it allowed us to sequence more rare strains, providing a more complete picture of the diversity existing in the hospital. We then combined the detected genomic diversity with the available metadata to examine potential differences between CA and HA strains and to investigate risk factors and phyletic patterns of genes and traits of interest.

## RESULTS AND DISCUSSION

### Data set summary and antibiotic resistance.

**FIG 1:**
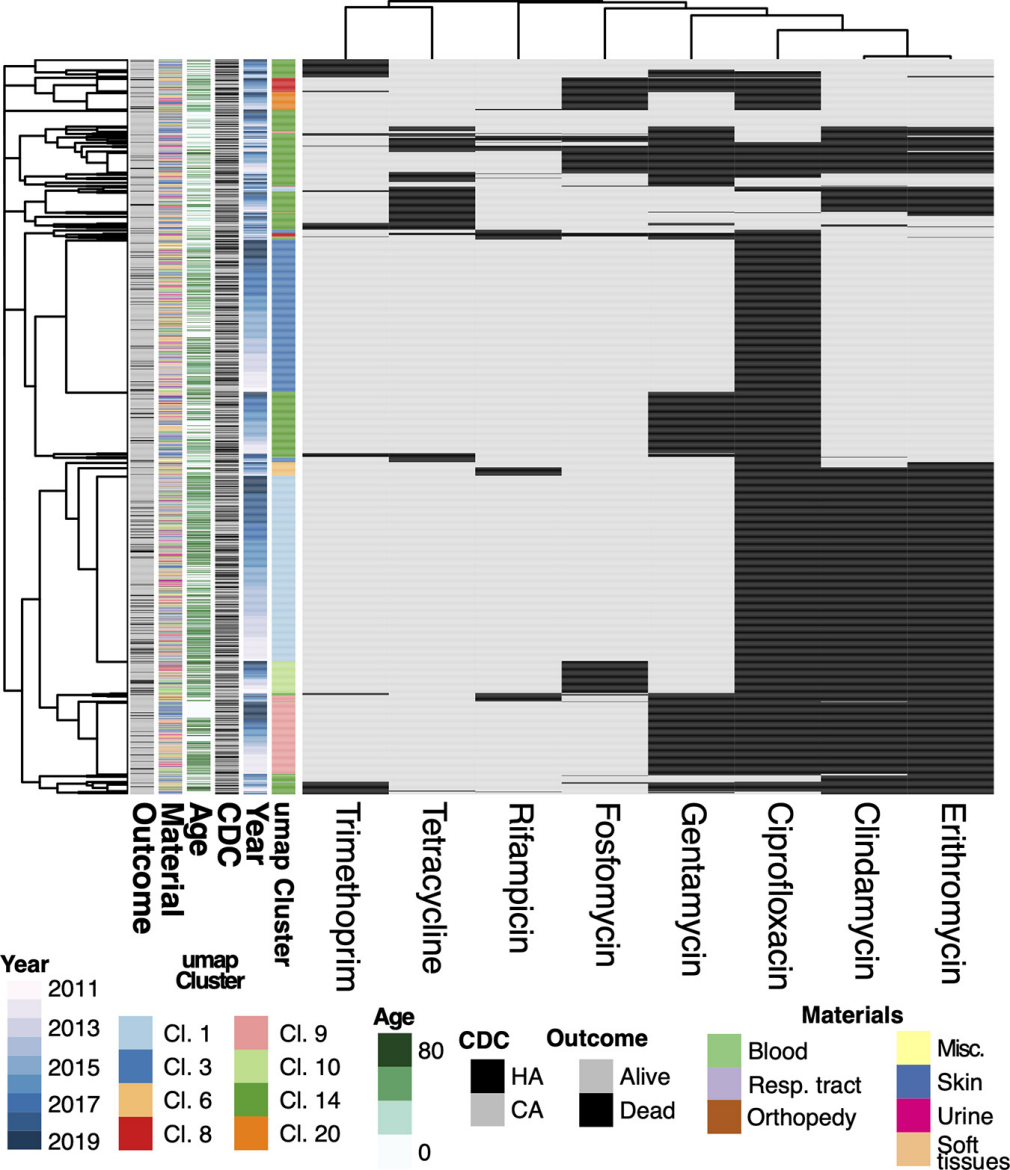
Summary of the MRSA samples grouped on the basis of similarities in resistance profiles for 8 antibiotics, chosen because the percentage of resistant strains varied between 5% and 99%. Black squares on the right indicate resistance, while gray squares indicate susceptibility. Additional metadata are shown in the six columns on the left, highlighting a relatively homogeneous distribution for outcome, material, age, CDC classification, and year of sampling with respect to the patterns of resistance.

Our data set comprises a total of 7,523 records from different hospital wards and clinical samples, from 5,233 patients who tested positive for S. aureus in 2011 to 2019. Of these patients, 2,807 stayed at the hospital at least overnight and are thus considered inpatients, while the remaining 2,426 were classified as outpatients. Of the 7,523 records, 1,404 (26.8%) are methicillin resistant (MRSA), a resistance that was more common in samples from inpatients (32.2%) than in outpatients (20.5%). MRSA isolates from inpatients were enriched (*P* < 2.2e-16) in blood (22%) and respiratory tract (22%) samples, while those from outpatients were more often from skin (39%) and soft tissue (35%) (see Fig. S1 in the supplemental material). Fig. 1 shows a clustering of the MRSA isolates based on profiles of resistance to the eight antibiotics for which the percentage of resistant strains varied between 5% and 99%.

Linear regression estimated MRSA isolates to have decreased from 34.8% in 2011 to 23.5% in 2019 (*P* = 0.0015). This change in percentage is not due to a reduction of MRSA in absolute numbers, but to the increase of yearly methicillin-susceptible S. aureus (MSSA) isolates, which in the analyzed time span increased from 73 in 2011 to 523 in 2019 (beta = 16.3, *P* = 0.002).

Resistance to penicillin, ciprofloxacin, erythromycin, clindamycin and gentamicin were the most common across both MRSA and MSSA isolates, with the former characterized on average by a larger number of resistances per isolate (Table S1). Resistance to teicoplanin, daptomycin, and linezolid were found in less than 1% of isolates (generally regarded as false positives; were not used for downstream analyses), while resistance to vancomycin was not detected. Linear regression highlighted temporal trends in the relative abundance of resistance. MRSA strains resistant to clindamycin, erythromycin, fosfomycin, ciprofloxacin, and rifampicin decreased in time, while the percentage of MSSA strains resistant to clindamycin and erythromycin increased significantly, and those resistant to penicillin decreased (Table S2).

### Selection of strains for sequencing.

To select strains for sequencing that captured the maximum amount of diversity, we reasoned that the antibiotic resistance profiles available for all isolates provides a valid proxy of genomic relatedness/divergence. On this premise, we clustered isolates based on resistance profiles and selected isolates from each cluster, while also maximizing the temporal spread. This aim was achieved through a two-step procedure: the uniform manifold approximation and projection (UMAP) algorithm was used to reduce the dimensionality of the antibiograms, and DBSCAN was used to partition observations into groups of similar profiles.

**FIG 2:**
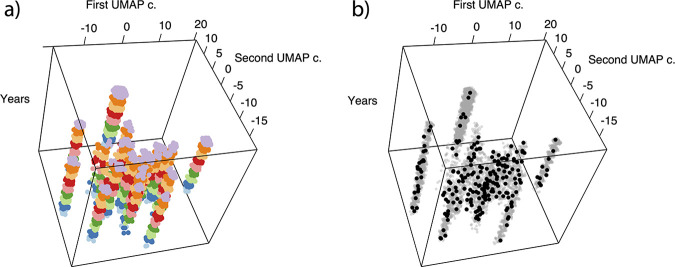
UMAP transformation of the data and integration with sampling year. The *x* and *y* axes correspond to the first two UMAP components and are therefore a function of the original resistance profiles, while the *z* axis corresponds to the year of sampling (*z* = 0 is 2011). (a and b) In panel a the colors indicate the sampling years (2011 to 2019), while in panel b the black dots indicate the isolates selected for genome sequencing.

This procedure resolved the 243 unique resistance profiles of the 7,523 isolates into 24 clusters (Fig. 2), 16 of which contained only MSSA isolates. Isolates were selected for sequencing to include, where possible, at least one isolate per cluster per year of study. This resulted in the selection of 226 samples, encompassing 79 different resistance profiles, including some below 1% frequency (see Table S3 for a detailed description of the resistance profiles and corresponding sequenced isolates). By comparison, random sampling resulted in a significantly smaller number of unique resistance profiles (49 ± 4).

### Genomic analysis.

The 226 genomes were sequenced and assembled (see Materials and Methods)*. In silico* multilocus sequence typing (MLST) identified 8 clonal complexes (CCs) and 49 sequence types (STs), including 16 novel ones STs 6438 to 6452 and ST 6461 that were submitted to PubMLST (28). We identified 87 Spa types and 4 staphylococcal cassette chromosome *mec* (SCC*mec*) types: classical nosocomial SCC*mec* types I (*n* = 11) and II (*n* = 6) represented a minority, while cassette IV, historically community associated (*n* = 74), made up 76% of the 97 MRSA isolates. SCC*mec* V, another community-associated cassette, was detected in only 6 genomes. A detailed description of the isolates and their genomic characteristics is given in Table S4.

The sampling strategy used allowed us to detect rare strains that otherwise would have remained unseen with a sequencing effort based on random sampling. Indeed, we found 37 locally rare STs (i.e., represented by one or two genomes in the entire data set), 16 of which are completely novel, for which we had to define novel STs. The phylogenetic tree of our entire data set shows that we sequenced genomes from 13 rare and phylogenetically divergent lineages, which based on branch length can be clearly seen as not closely related to the common ones (Fig. S2).

Although our sampling strategy intrinsically prevents the use of genomic data in conjunction with information regarding the relative frequencies of the resistance profiles, this approach maximizes phylogenetic breadth and thus allows us to investigate how often virulence and antibiotic resistance genes (ARGs) are lost or gained over evolutionary time. ARGs impose a fitness cost in the absence of the antibiotic, and this may provide strong selective pressure for their rapid loss after treatment (29). We explore these dynamics using the phylogenetic signal (here indicated by D) as previously implemented in R (30) (Table S7). Briefly, this metric provides a means to gauge the concordance between the presence/absence of a given trait with the branching pattern of the underlying tree; a negative value reflects high concordance, while a positive value indicates independence from the phylogenetic topology and, therefore, dispersion of a trait. A trait in this context can be a gene, a phenotype, or any other binary feature (presence/absence) associated with the isolates. As a control, we calculated the same metric for a trait that is, by definition, highly consistent with the tree, i.e., belonging to a clonal complex (CC22).

The D value of all-cause mortality in this analysis is 1.5, indicating a very low correlation of this trait with the phylogenetic tree. This suggests that the outcome of S. aureus sepsis could be largely independent from the S. aureus lineages and that other factors are likely accountable for the death of the patients (such as starting health condition of the patient, nature of the illness/wound, age). This topic was further explored with the *ad hoc* analysis presented below.

The presence/absence of all virulence factors is consistent with the underlying tree (D < –1), suggesting identity by descent to be the dominant mode of evolution, rather than frequent horizontal transfer. Genes linked within a single pathogenicity island (e.g., *splA* and *splB*) show highly consistent results, as expected for linked genes inherited together.

D values for antibiotic resistance genes are also mostly negative, but closer to zero than those for virulence genes, suggesting a faster rate of gains and losses, which is to be expected, as many resistance genes are carried on plasmids. We speculate that larger or positive D values for antibiotic resistance genes reflect variable levels of antibiotic exposure, combined with strong selection pressures for the gain and loss of these genes. Resistance genes may impose a fitness cost in the absence of the antibiotic, and the plasmid carrying these genes can be lost quickly if compensatory mutations are not acquired. Alternatively, resistant and sensitive strains may coexist in the environment.

Rifampicin and ciprofloxacin show extreme values; the former is the only character highly over dispersed on the tree, which is consistent with very frequent losses and gains (D = 1.86). In contrast, ciprofloxacin resistance is by far the most stable (D = –20), which is consistent with previous findings (31). While both these resistances are due to mutations in housekeeping chromosomal genes, there are important differences between them. Resistance to rifampicin is typically caused by single mutations in the *rpoB* gene (32), while resistance to ciprofloxacin requires multiple mutations in different genes (mainly in genes encoding DNA gyrase and topoisomerase IV, the targets of the antibiotic) (33). Additionally, mutations conferring resistance to rifampicin are associated with a significant fitness cost in the absence of antibiotic, while mutations conferring resistance to ciprofloxacin confer a comparatively mild decrease in fitness (29, 34). Both the fitness cost and the resistance level can be reduced by additional mutations at the same or different loci in the absence of antibiotic pressure (35).

We conclude that resistance to rifampicin is easy to develop but costly to maintain, while resistance to ciprofloxacin is more difficult to acquire (multiple mutations required) but less expensive. Indeed, mathematical modeling has previously shown that even small differences in fitness can translate into very different survival times after removal of selective pressure (e.g., 36). According to the modeling by Nowak, if rifampicin-resistant strains have a 25% decrease in fitness with respect to ciprofloxacin (29, 34), they will be replaced more than four times faster by wild-type strains once antibiotic treatment is removed.

**FIG 3:**
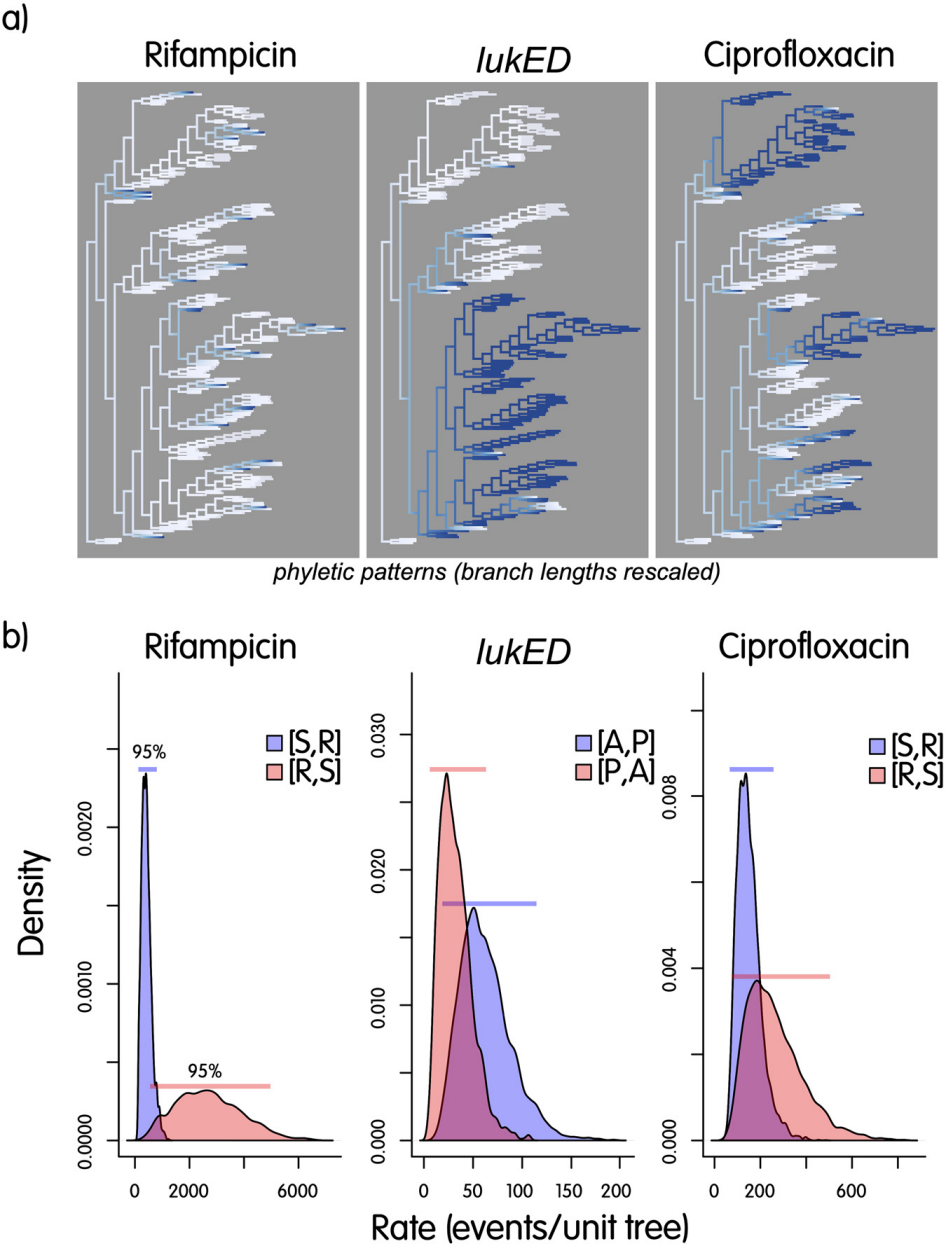
(a) Phyletic patterns with ancestral state reconstruction for two resistance phenotypes and one virulence gene. Rifampicin resistance is highly scattered across the tree and is lost quickly, consistent with a high fitness cost in the absence of the antibiotic. This contrasts with the ciprofloxacin resistance, which is more common and carries a lower fitness cost. The distribution of *LukED* closely matches the phylogeny. (b) Evolutionary rates for the same traits, indicating that reversion rates (from resistant to sensitive) for antibiotics are on average higher than the rate of loss of virulence genes. The trees have branch lengths rescaled for graphical purposes.

Additionally, we performed a birth-death analysis to explore the relative rates of gains and losses of these two resistance traits compared to the presence/absence of a virulence gene, *lukED* (see Materials and Methods; Fig. 3b). This confirmed that resistance tends to be lost more commonly than gained, while the opposite is true for the more stable virulence gene, *lukED*. Rifampicin resistance is more rapidly lost than ciprofloxacin resistance, which again is consistent with the former conferring a greater fitness cost.

### Community- and hospital-acquired infections.

S. aureus infections are usually defined as hospital or community acquired (HA and CA) based on epidemiological criteria. While most early studies suggested genetic characteristics that may differentiate HA and CA, later comparative studies have challenged this view (14).

According to the CDC epidemiological criteria, out of the 2,807 inpatients positive for S. aureus in our data set, 51.7% are classified as CA (while the 2,426 outpatients were clearly all classified as CA). HA isolates were more common in blood (27%) and respiratory tract samples (29%), while CA isolates were more commonly recovered from skin (24%) and soft tissue infections (33%) (Fig. S1). During this period, CA infections increased in yearly numbers (from 39 in 2011 to 182 in 2019), while HA remained stable, leading to an increase of CA from 38% to 54.4% of the total (linear regression *P* < 0.01, beta = 1.8), becoming the primary source of infections in the hospital. By additionally partitioning HA and CA infections into MR and MS, we observed significant changes in the prevalence of CA-MSSA and HA-MRSA in inpatients. The percentage of CA-MSSA increased from 27% to 42.1%, while HA-MRSA declined from 30.2% to 16% (see Table S5 for raw numbers and regression values). Comparison of resistance patterns of HA and CA samples indicates that the former tend to have more resistances than the latter, with the largest differences being for methicillin (37.89% and 27.05%, respectively) and ciprofloxacin (39.29% and 27.8%, respectively) (Table S6). However, since MRSA and MSSA are nonhomogeneously distributed in the two groups, we explored how the number of different antibiotic resistances is distributed across the four groups, CA-MSSA, HA-MSSA, CA-MRSA, and HA-MRSA (Fig. S3), confirming that the main differences reflect the MRSA/MSSA partition. As expected, and previously reported (20), isolates sensitive to all or most antibiotics are more often MSSA, while multidrug-resistant isolates tend to be MRSA (Table S2).

Considering the 226 sequenced genomes of sepsis isolates, 35% were HA and 65% CA. We checked for the presence of markers known to be associated with CA, such as the SCC*mec* type and the presence of the PVL gene. However, no trait was found to be significantly associated with either HA or CA strains, confirming previous findings that showed that the two epidemiological classes are not distinct at the genomic level.

### Mortality rate.

Through the studied period, S. aureus infections can be connected to at least 435 all-cause deaths (8.3%; mean age, 71.8 years), 418 of which were inpatients. We used generalized linear models (GLM) to identify predictive factors of mortality. At first, we used the whole metadata associated with samples and patients, including antibiotic resistances, site of infection/sampling, and age as predictors (Table S7). This resulting model highlighted that the most significant predictor associated with fatal outcome is age, together with two source materials, blood and respiratory tract, which are associated with invasive infections and higher mortality rates (42.5% and 30%, respectively). Two antibiotic resistances, to methicillin and fosfomycin, are also positively significantly associated with mortality. Resistance to ciprofloxacin is associated with increased survivability. Mortality rate due to MR infections was higher that due to MS infections (15% to 5.9%), a difference that may be associated with the higher average number of resistances in MR isolates (Fig. S3). Additionally, the average length of stay (LOS) for MR infections is significantly longer than for MS infected inpatients (31.2 and 18.21, respectively). Older patients usually have weakened immune systems, which makes any infection potentially lethal; this might mask the smaller contribution of other predictors. For this reason, we also built a model removing isolates from patients more than 60 years old (Table 1). This model confirms the one above and additionally identifies urine infections as leading to an increased risk of death.

**TABLE 1:** Significant (*P* < 0.05) predictors of worse outcome when patients older than 60 years are removed from the analysis and the predictors indicated in Materials and Methods^*a*^

Predictor	Estimate	SE	Z value	Significance
Intercept	−4.77	0.85	−5.59	2.22E-08
Methicillin	1.62	0.70	2.32	2.02E-02
Fosfomycin	1.90	0.84	2.25	2.43E-02
Ciprofloxacin	−1.73	0.76	−2.28	2.25E-02
Blood	2.69	0.68	3.97	7.09E-05
Respiratory tract	3.07	0.68	4.51	6.42E-06
Urine	2.41	0.98	2.45	1.43E-02

a This filtered data set highlights additional predictors that may be important when infections involve younger patients.

Lastly, the mortality of CA and HA strains was 13.2% and 16.7%, respectively. For the latter, we report a significant mortality reduction (*P* = 0.03, beta = –0.7), from 20.5% in 2011 to 13.1% in 2019, while casualties associated with CA strains were relatively stable (standard deviation = 2.2%). By exploiting the additional MR/MS partitioning, we detected a significant (*P* = 0.02, beta = –0.4) decline over time in the number of HA-MR casualties.

When focusing on the isolates selected for genomic sequencing, the overall mortality rate was 29.7% (*n* = 53). The attributable mortality rate for MSSA (*n* = 29) and MRSA (*n* = 24) blood infections was 28.7% and 31.1%, respectively. None of the CCs were found to be significantly associated with an increased risk of death, even though we found CC22, CC8, and CC30 to have a rate of mortality above the mean, standing at 40.9%, 39.3%, and 33.3%, respectively.

This result agrees with that described above, with the distribution of mortality on the phylogenetic tree being best explained by a model that does not account for phylogenetic structure (Table S8). The two results indicate that mortality is more dependent on characteristics of the host/infection site than on genomic properties of the specific S. aureus strain. To explore this issue in more detail, we applied phylogenetic regression to mortality using as predictors both hospital and genomics data. In contrast to the generalized models described above, in this case we integrated the structure of the phylogenetic tree to take into account the nonindependence of the observations. The model identifies several predictors that are significantly associated with the outcome; the most important is age, followed by contracting the infection once inside the hospital (CDC-HA). We also identified some antibiotic resistances and virulence factors that are associated with the outcome (Table S9).

It must be noted that our analysis presents limitations. First, we were not able to evaluate the association between genotype and important clinical outcomes outside mortality (e.g., readmission, prolonged bacteremia), as we had no access to these metadata. Additionally, our numbers are limited; this could explain discrepancies with previous studies suggesting a significant relationship of genotype and mortality (e.g., 37, 38). It should be considered, however, that studies such as those cited above use multivariate regression without taking into account the fact that genomes are not independent observations. This makes all studies where this is not considered potentially flawed. The major technical novelty of our paper is indeed that we account for phylogenetic distances in the regression analysis, which enables us to remove the confounding effect of an uneven sampling of the different CCs. We posit that future studies, with higher numbers, following our unbiased sampling approach and considering genomes as not independent observations could be the key to better understanding whether and how much genotypes and outcomes can be associated.

### Clonal complexes.

**FIG 4:**
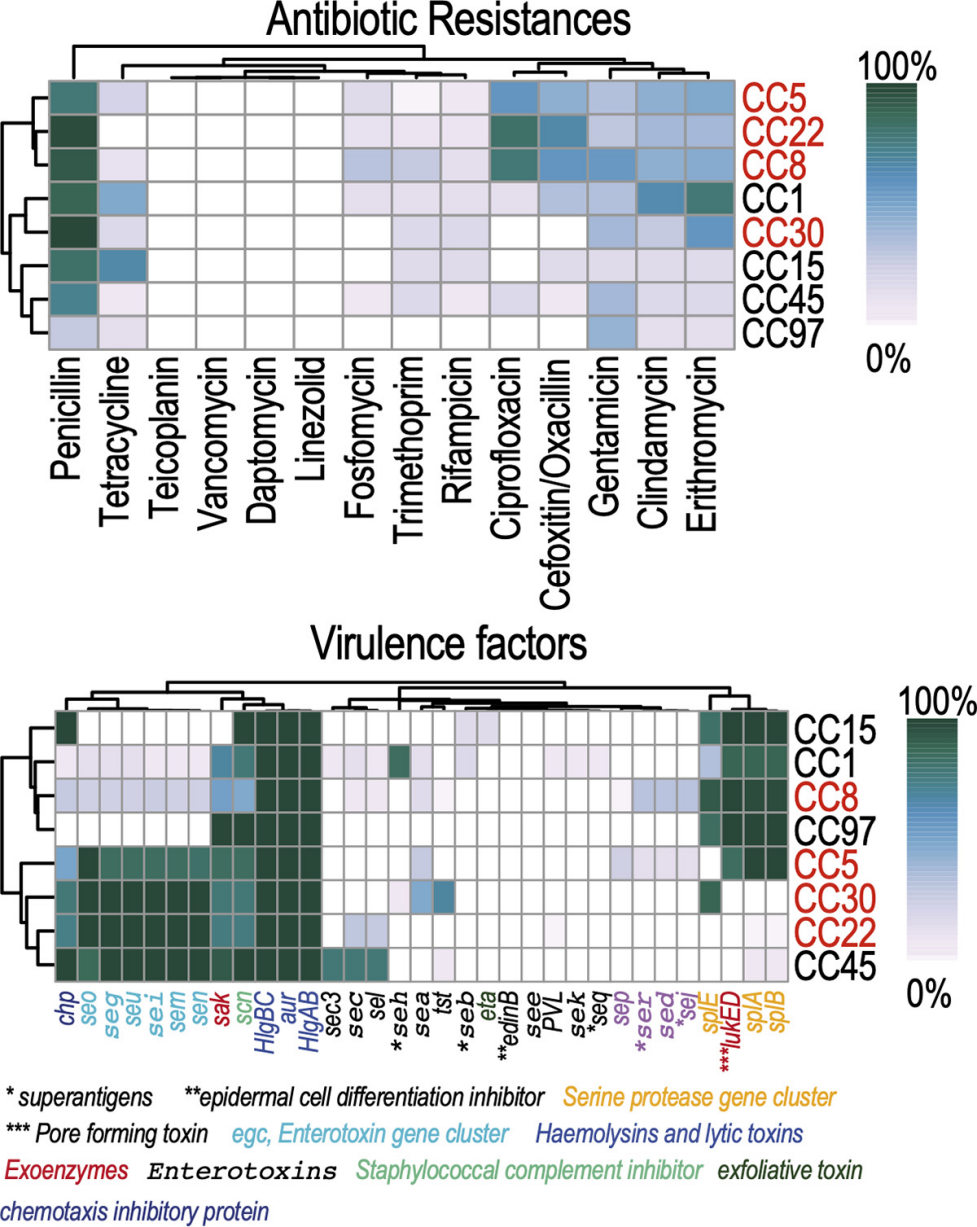
(a and b) Summary of antibiotic resistances (a) and virulence factors (b) in a clonal complex perspective. Cells in the heatmap correspond to the frequency of strains possessing a resistance or virulence factor in a given CC. Functional annotation of virulence genes is also indicated.

Almost half (*n* = 109) of the 226 sequenced strains belong to CC5, CC8, and CC22, which are known to be globally abundant, and we therefore inspected them in more detail, together with CC30, a complex characterized by a high all-cause mortality rate (33%). We used core single-nucleotide polymorphism (SNP)-based phylogenetic analysis and profiling of virulence and antibiotic resistance genes, and the results are summarized in Fig. 4 (see also Fig. S4 and S5 for trees without and with branch lengths). Figure S6 shows the heatmaps of the SNP distances between pairs of isolates, highlighting the presence of clusters of genomes with high levels of similarity.

The phylogenetic analyses, which include genomes from public database of the Bacterial and Viral Bioinformatics Resource Center (BV-BRC) (39) (see Table S10), provide the means to determine whether monophyletic clades of closely related genomes can be found in our data set. Indeed, such clades can be spotted in the phylogenetic tree of CC8 and CC22 and encompass isolates dating across the 9 years of our data set, indicating that these strains are endemic to the hospital. The phylogenetic tree of CC5 is also characterized by several small monophyletic clades of highly related genomes, but these are more limited in time, indicating that there were independent introductions in the hospital with consequent outbreaks that were quickly eradicated. The phylogenetic tree of CC30 reveals interesting differences with respect to the other three CCs: genomes are more diverse and spread on the tree with no monophyletic clades, suggesting sporadic but deadly infections, with no strain apparently persisting in the hospital. This lower persistence of CC30 could be explained by an average lower resistance to antibiotics of these isolates (Fig. 4), as also previously reported by Aanensen and colleagues (20).

Besides being resistant to penicillin, and less commonly to tetracycline, CC5, CC8, and CC22 are also usually resistant to ciprofloxacin and oxacillin/cefoxitin, these two being rare in other CCs, and to a lesser degree also gentamicin and erythromycin, which are shared with several other CCs (Fig. S5a). Isolates belonging to CC30 show much lower levels of antibiotic resistance. CC5, CC22, and CC30 possess the enterotoxin gene cluster (*ecg*, light blue in Fig. S5b), genes that are only sparsely present in other CCs, except for CC45. They also code for hemolysins, chemotaxis inhibitory protein (Chp), and staphylococcal complement inhibitor (Scn). CC5 and CC8 show the sporadic presence of several additional toxins (light purple in Fig. S5b) that are not found in any of the other CCs, including some that were annotated as superantigens (*ser* and *sel*) and share the presence of a serine protease operon (represented here by the *splABE* genes) together with the pore-forming toxin gene *lukED*. A portion of CC30 isolates also harbor *tst*, an association consistent with previous genomics studies (20). This gene has been implicated in toxic shock syndrome in susceptible hosts (40). This serious and often fatal condition is triggered by superantigen toxins causing a cytokine avalanche by T cells manifesting as fever, rash, shock, and rapid and multiple organ failure (41).

**Conclusions.** In this work we present a strategy for maximizing the diversity of clinical strains that can be characterized through genomics in real-world settings with finite resources. The strategy exploits antibiograms as a proxy of genetic diversity to select isolates for sequencing, as these data are usually generally available for all isolates in clinical settings. We validated our strategy on an S. aureus collection, demonstrating how it can reveal rare lineages by reducing the redundancy that characterizes bacterial populations, especially in hospitals, where outbreaks of identical clones are common.

This approach provides a means to avoid the oversampling of very similar isolates from common STs, allowing us to obtain genomes from rarer strains, which would be less likely to be detected through a random population snapshot. We posit that obtaining the genomes of rare strains provides a more complete genomic and phylogenetic picture of the epidemiology of the hospital. This, in turn, grants advantages such as additional power to forecasting strategies, which can benefit from the early detection of rare strains that could be rising in prevalence and cause outbreaks in the future.

## MATERIALS AND METHODS

### Determination of antimicrobial susceptibility by MIC.

Staphylococcus aureus isolates from patients admitted to Fondazione IRCCS Policlinico San Matteo Hospital (Pavia, Italy) are routinely investigated by the automated system Phoenix (Becton, Dickinson, Franklin Lakes, NJ, USA) using PMIC-88 panels for the detection of antimicrobial resistances and determination of MIC values. MICs are interpreted with the EUCAST breakpoints (42) to define which strains are susceptible, intermediate, or resistant to each antibiotic. This work collects and exploits available metadata collected in the hospital for all the isolates of S. aureus and the respective patients, from 1 January 2011 to 31 December 2019. For all isolates, resistance profiles were collected with respect to 15 antibiotic compounds of clinical relevance: penicillin, teicoplanin, clindamycin, daptomycin, cotrimoxazole, oxacillin, cefoxitin, erythromycin, linezolid, rifampicin, vancomycin, gentamicin, fosfomycin, ciprofloxacin, and tetracycline. Oxacillin and cefoxitin results are used to define methicillin resistance. For all patients, the following data were retrieved and used: isolation material, patient age, admission date, outcome. It should be noted that due to technical issues occurring within the hospital servers/digital infrastructure before the beginning of this project, for the years 2011 and 2015 an unspecified number of samples were lost.

### CDC classification.

Community- and health care-acquired infections are currently defined based on criteria provided by the Centers for Disease Control and Prevention (CDC) (11). An isolate is considered CA if the patient fulfills the following criteria: (i) testing positive for an infection within 48 h of admission, (ii) not having undergone surgery in the 48 h since admission, (iii) not residing in a long-term-care facility before admission, and (iv) not having undergone hemodialysis/peritoneal dialysis in the past year or during the current admission. Instead, it is considered HA if a subsequently positive wound culture was taken after 48 h from the hospital admission or if one or more of the CA criteria are not met.

### Antibiogram-based isolate selection for genome sequencing.

All antibiograms with missing MICs, MICs classified as intermediate, or ambiguous MIC values were removed from the data set. The remaining MIC values were coded into binary variables R (resistant) and S (susceptible). Data set dimensionality was first reduced with a technique called uniform manifold approximation and projection (UMAP) using the R package uwot (43). Subsequently, DBSCAN enabled the identification of compact clusters in UMAP coordinates using the R package DBSCAN (44). This analysis informed the selection of 226 S. aureus isolates that capture all the cluster diversity.

### DNA extraction and sequencing.

DNA was extracted from 226 blood isolates using the DNeasy blood and tissue kit (Qiagen, Hilden, Germany), following the manufacturer’s recommendation and adding an initial step of incubation of the bacterial pellet with lysozyme (20 mg/mL) for 2 h; gel electrophoresis (Tris-acetate-EDTA [TAE], 1.5% agarose) was used to check DNA quantity and quality. Whole-genome sequencing was performed in paired-end mode (2 × 150 bp) on a NovaSeq machine (Illumina). Reads were quality checked with FastQC (www.bioinformatics.babraham.ac.uk/projects/fastqc/) and assembled with SPAdes v.3.14.1 (45), and quality was assessed with assembly-stats (github.com/sanger-pathogens/assembly-stats). In order to evaluate the completeness of our genome assemblies, we performed a previously described check (46). Briefly, we searched for the presence of core genome MLST (cgMLST) genes (determined in reference 47) using BLAST and verified that all genomes contained more than 95% of them (Table S4).

### *In silico* typing and annotation.

*In silico* multilocus sequence typing (MLST) was performed by comparing our genomes with the S. aureus MLST database (PubMLST) (28), using an in-house python script. Typing of the staphylococcal cassette chromosome *mec* (SCCmec) element was done with the web implementation of SCCmecFinder (48) with the options min_coverage = 80 and percentage identity = 90. Spa types were assigned with Ridom SeqSphere+ (48, 49). Presence/absence profiles of 32 virulence factors, chosen because they are commonly associated with bloodstream infections and involved in different clinical syndromes, were identified with VirulenceFinder v.2.0 (50), with the settings min_coverage = 80 and percentage identity = 90.

### Statistical analyses.

As recommended by the Clinical and Laboratory Standards Institute (CLSI) guidelines M39-A4 (51) and many recent papers (e.g., 52), statistical analyses were performed on the first isolate per patient only. R (v3.2.4) was used for all statistical analyses. Antibiotic resistance data were analyzed using the chisq.test function, while population proportions were assessed with the proportion test (Z test) through the function prop.test. The lm function was used for linear regression using time as a predictor; a significant regression coefficient (beta) for time was taken as an indication of a temporal trend in the data. For generalized models, we used the glm function and a combination of numerical and categorical variables as detailed in Results and Discussion. For models with outcome as the dependent variable considering all isolates, we included antibiotic resistance profiles, age, material, and CDC classification as predictors.

### Phylogenetic analyses.

A core SNP-based phylogeny and an SNP distance matrix were generated for each of the selected clonal complexes (CC) using the P-DOR pipeline (53). Briefly, the pipeline contextualizes the query genomes with background data sets, based on genetic similarity. Core SNPs are then called, aligning the genomes to a reference. The following reference genomes were used: GenBank accession no. NC_017763.1 (CC22), BX571856.1 (CC30), CP026068.1 (CC8), and CP021105.1 (CC5); the BV-BRC (39) genomes used for contextualization are available in Table S10. The resulting core SNP multialignment was fed to IQ-TREE (54) to obtain phylogenetic trees for each CC. P-DOR was also used to generate the phylogenetic tree of the entire novel local data set (GenBank accession no. BX571858), skipping the step of retrieval of database genomes from BV-BRC (39).

We estimated the transition rates of a simple birth-and-death model based on the phyletic pattern of interest, whose states are given by sensitive (or absent, S or A) and resistant (or present, R or P) and are on the core SNP-based phylogenetic tree (Fig. 3b). The mcmcMk function from the R package phytools (55) applied here uses the tree and the phyletic pattern to estimate the transition rates among the two states and was run for 50,000 generations; the function contMap from the same package was used to reconstruct and map ancestral states on the phylogenetic tree; the phylogenetic signal was estimated using the function phylo.D from the package caper (https://rdrr.io/cran/caper/) and 1,000 permutations. Linear phylogenetic regression was performed with phylolm (56) to explicitly consider the nonindependence of observations through the relationships summarized by the phylogenetic tree. Before using presence/absence profiles of virulence genes as predictors in phylogenetic regression, we removed colinear profiles that are known to affect parameter estimation in a negative way. This was done by identifying genes with profiles 90% identical or more and then using one of the profiles as a representative in regression, resulting in 18 virulence gene patterns over 26 genes.

### Data availability.

The genome sequences are available at NCBI under BioProject accession number PRJNA797572. Accession numbers of BV-BRC genomes used for contextualization are listed in Table S10.
