# Supplementary figures and images for: Combining Genome Surveillance and Metadata To Characterize the Diversity of Staphylococcus aureus Circulating in an Italian Hospital over a 9-Year Period
Source: Microbiol Spectr. 2023 Jul 17;11(4):e01010-23. doi: 10.1128/spectrum.01010-23 (PMC10433831; doi:10.1128/spectrum.01010-23)

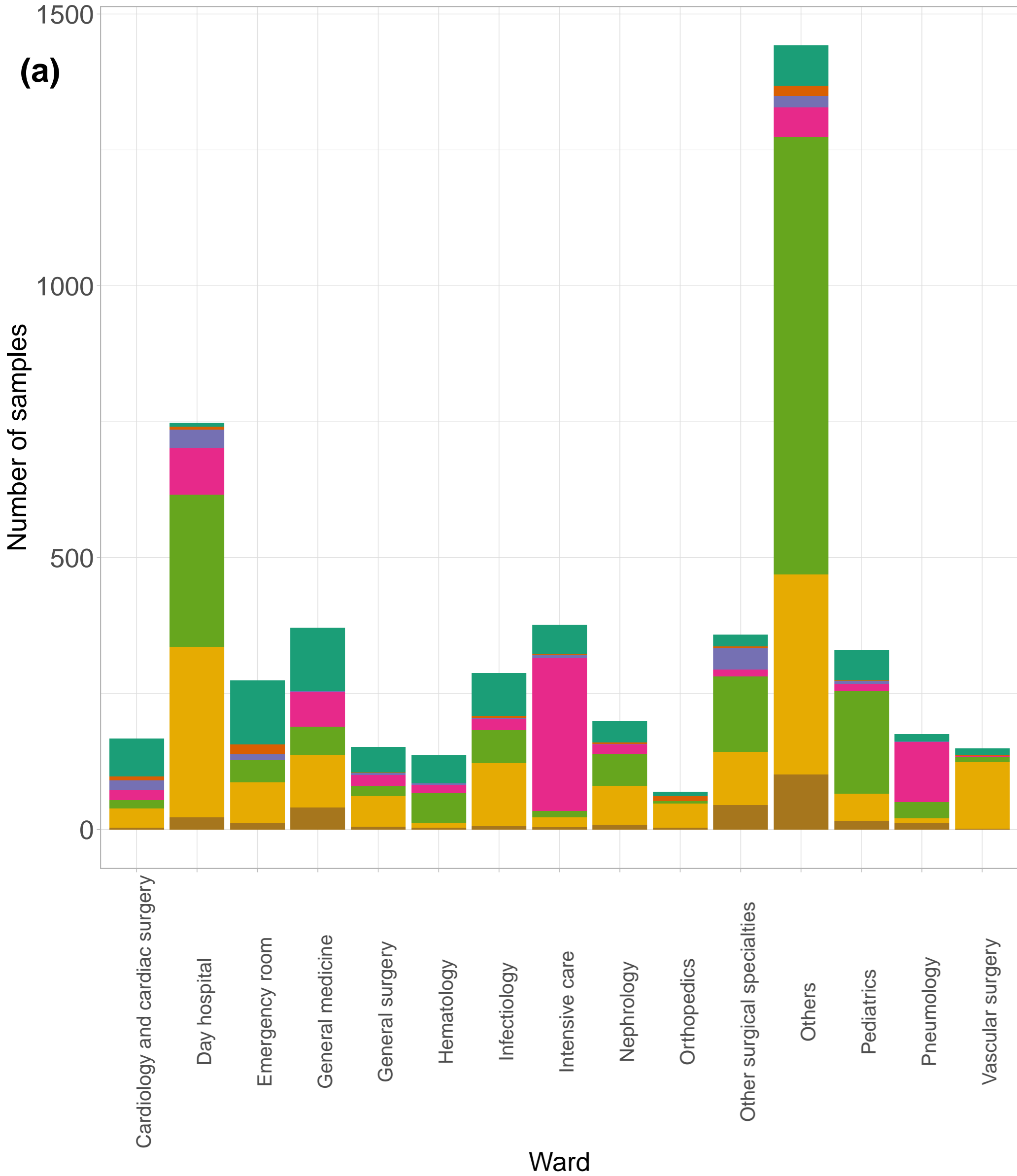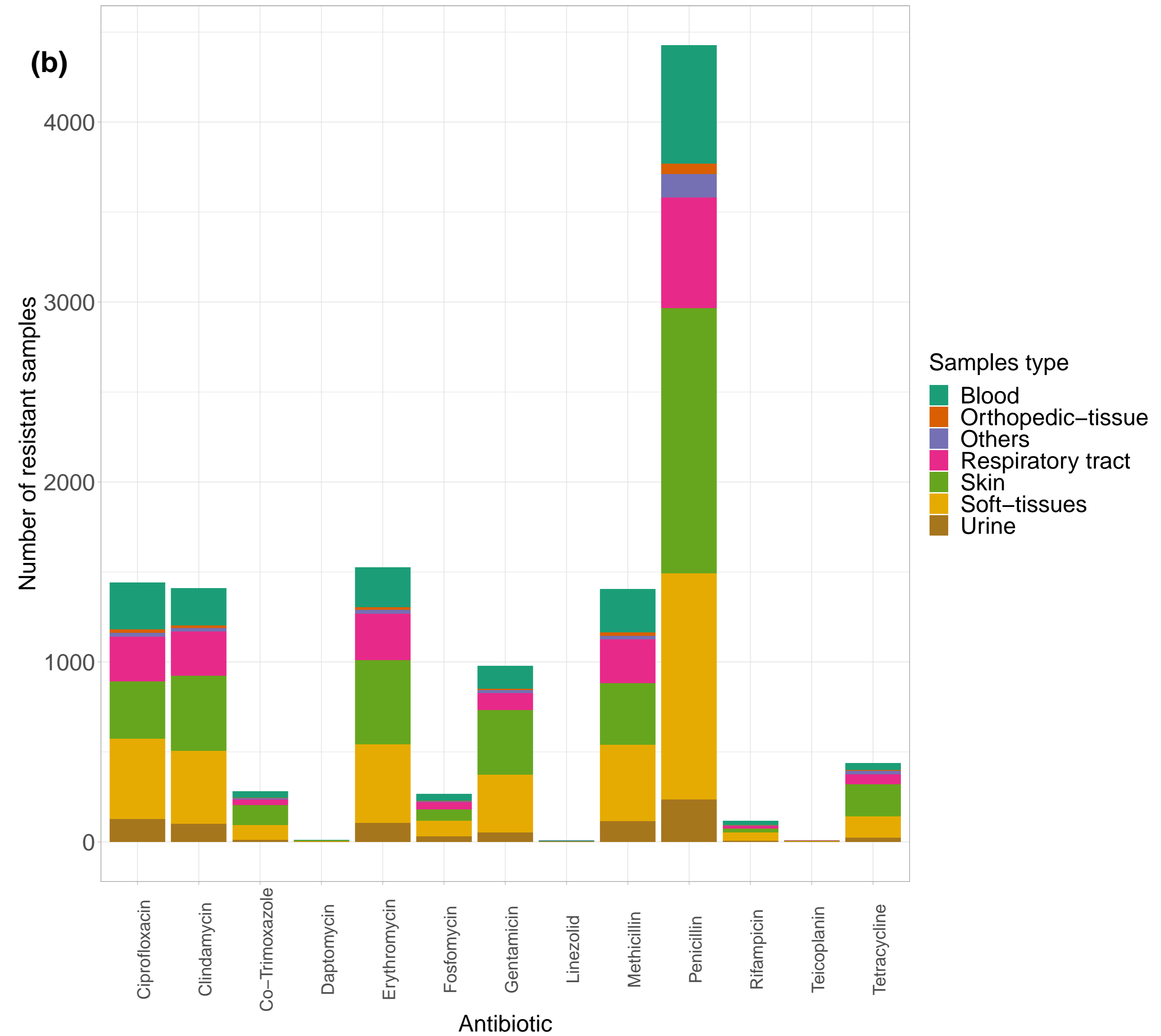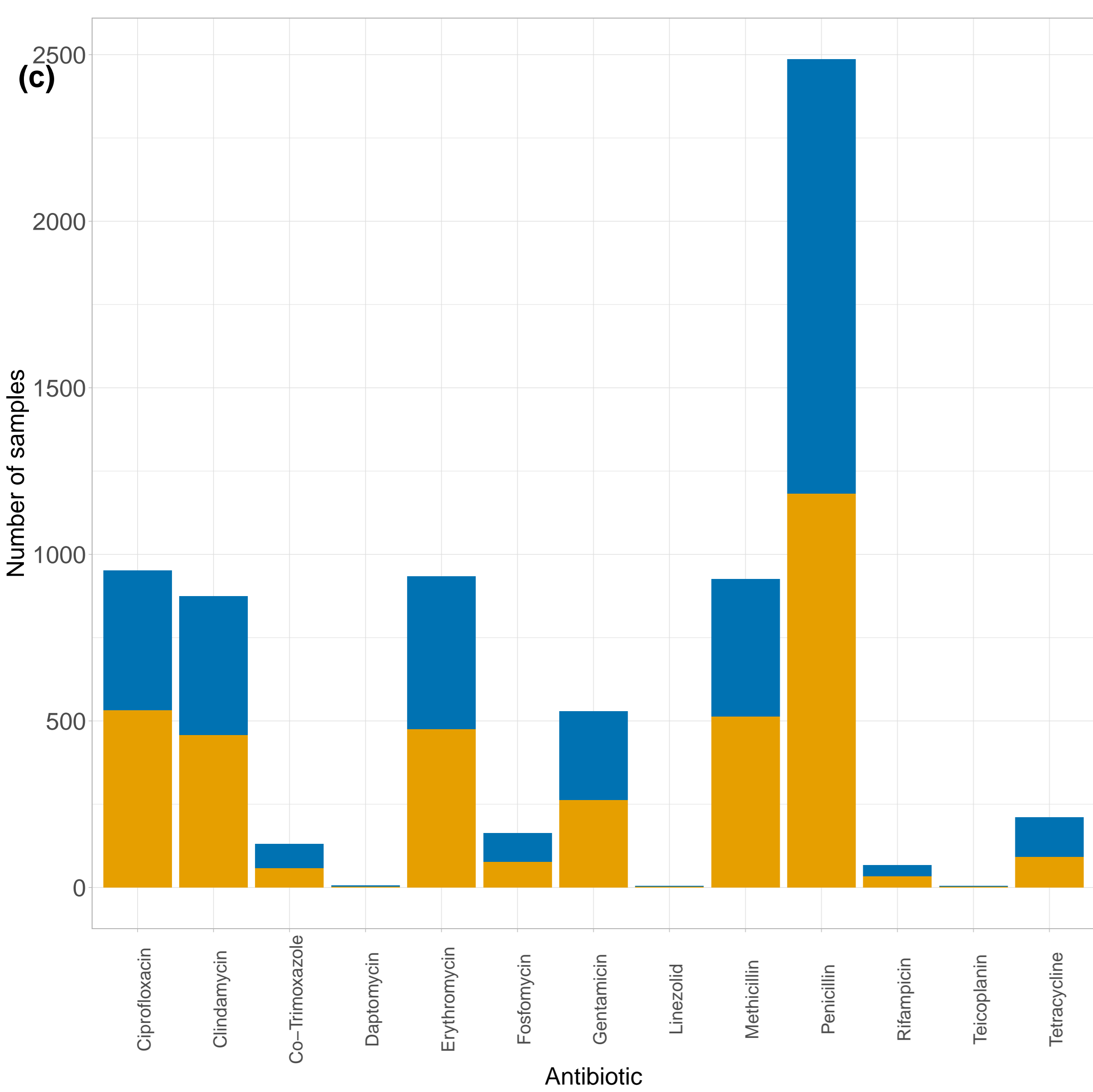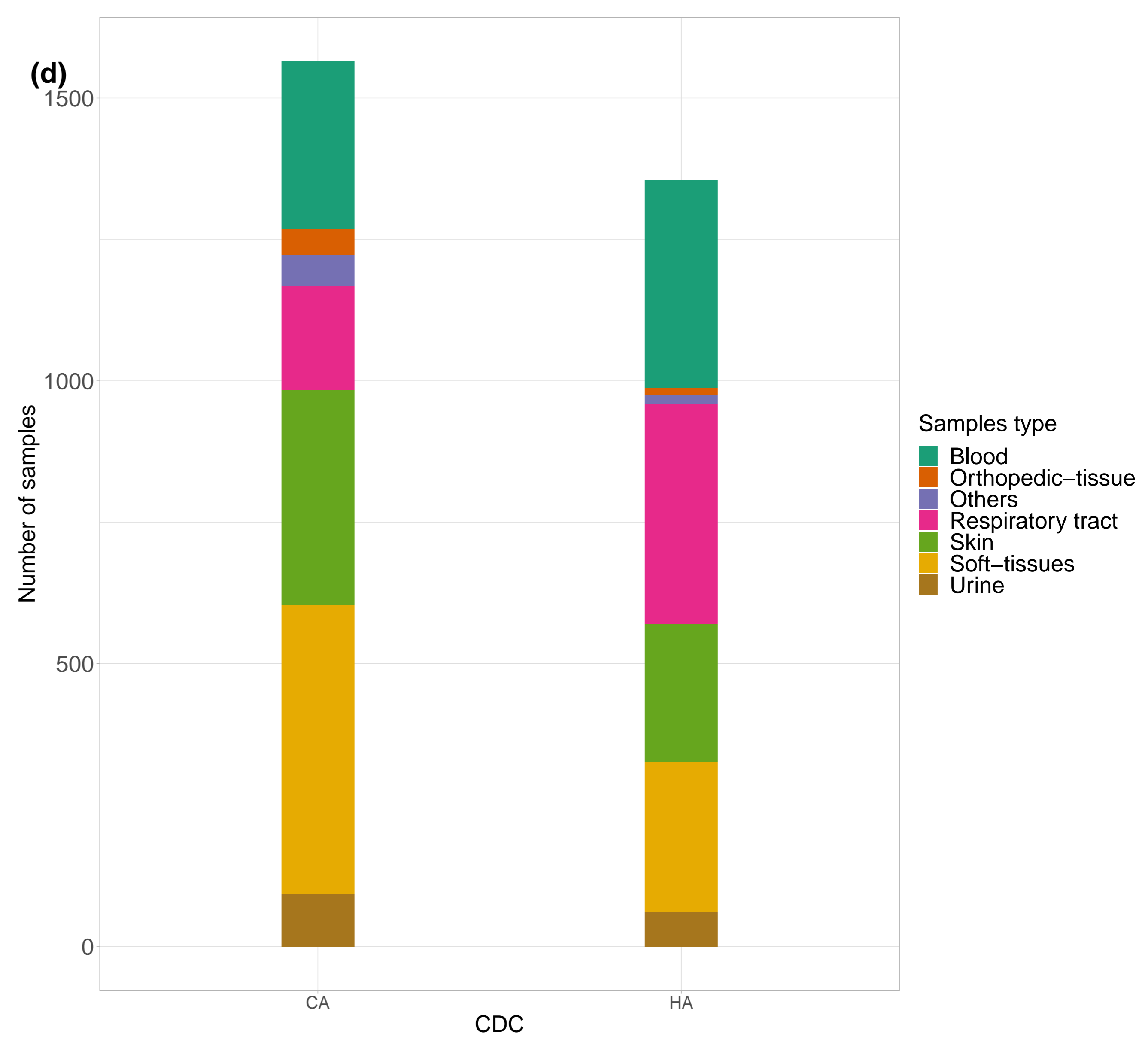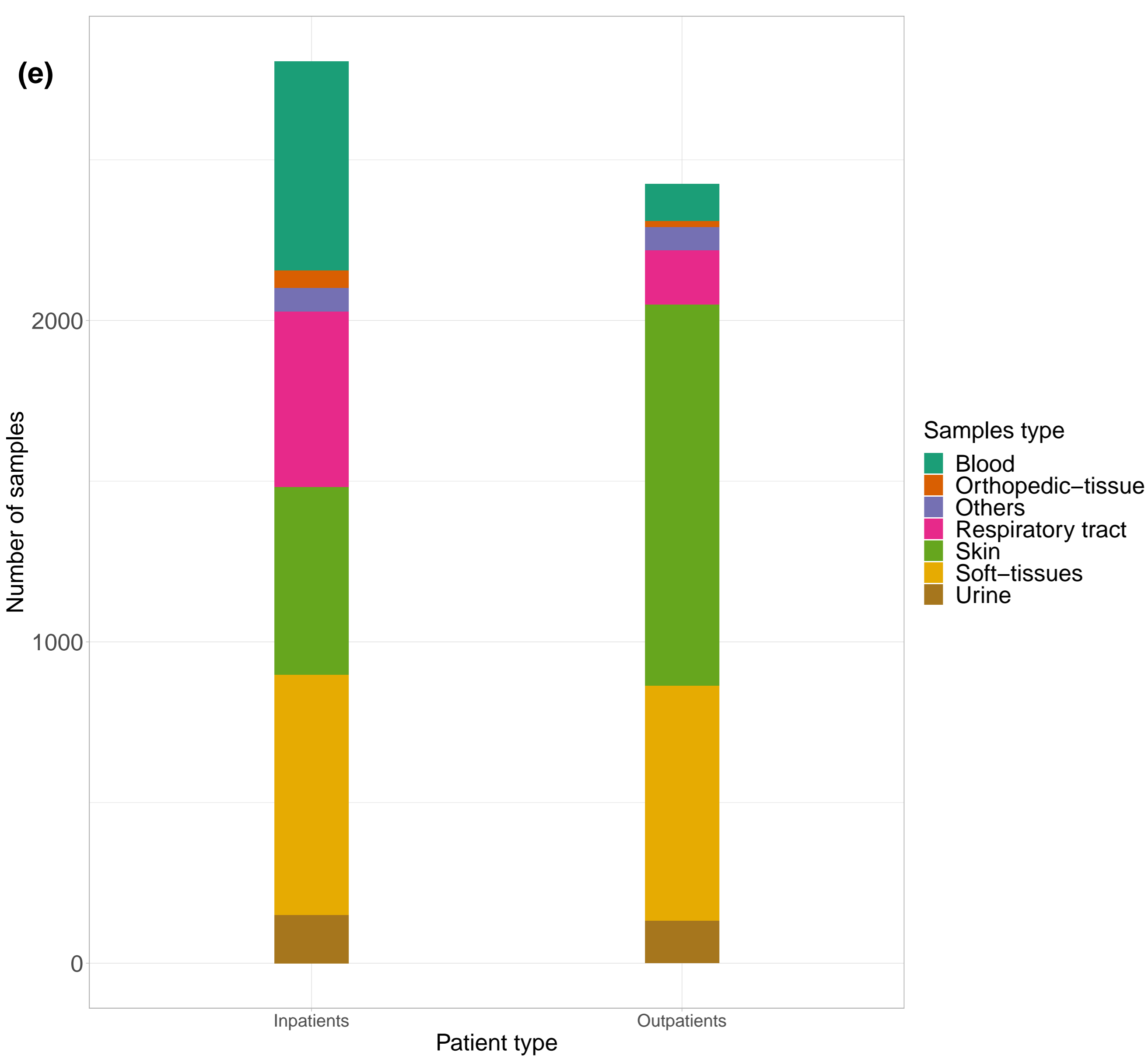

Supplement: Supplemental file 1 — Figure S1. Download spectrum.01010-23-s0002.pdf, PDF file, 0.01 MB [file spectrum.01010-23-s0002.pdf]

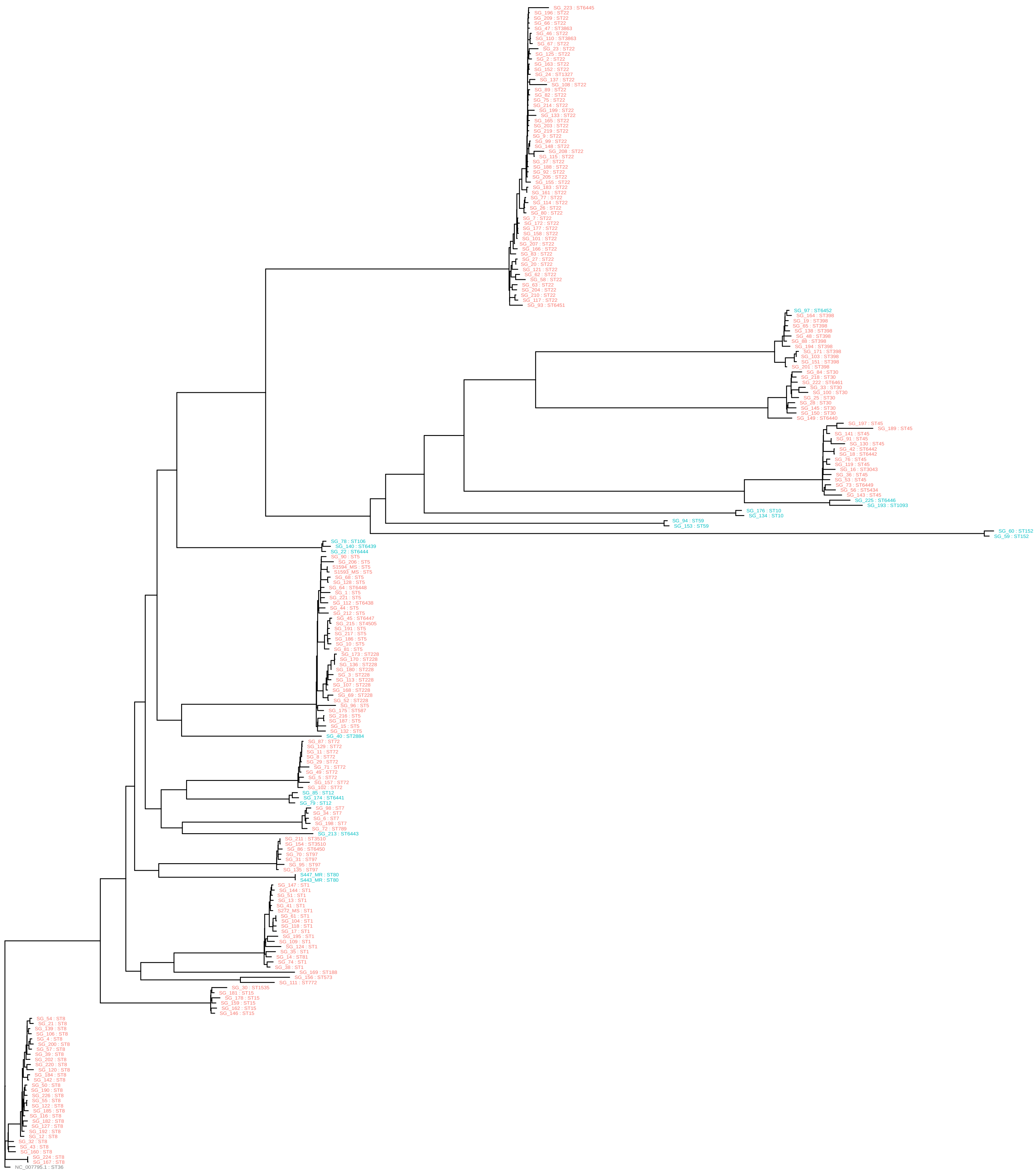

major  
rare

Supplement: Supplemental file 3 — Figure S3. Download spectrum.01010-23-s0004.pdf, PDF file, 0.02 MB [file spectrum.01010-23-s0004.pdf]

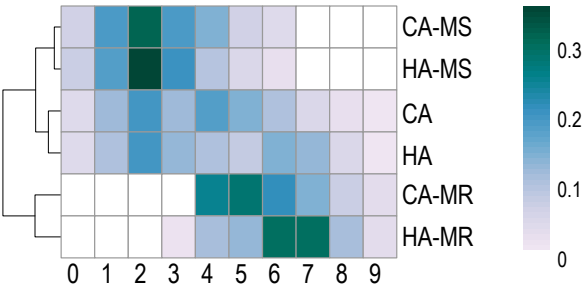

Supplement: Supplemental file 4 — Figure S4. Download spectrum.01010-23-s0005.pdf, PDF file, 0.07 MB [file spectrum.01010-23-s0005.pdf]

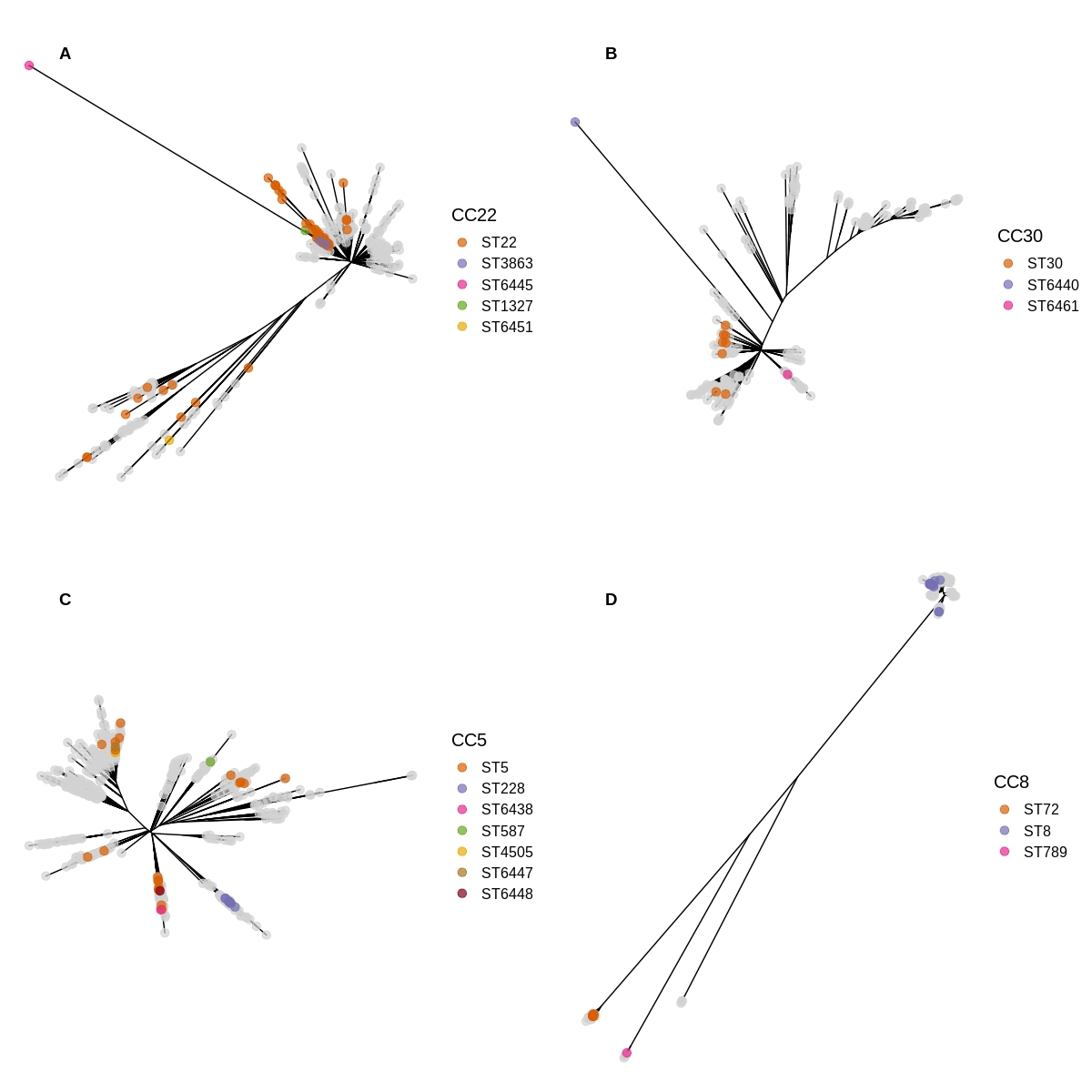

Supplement: Supplemental file 5 — Figure 5. Download spectrum.01010-23-s0006.jpg, JPG file, 0.2 MB [file spectrum.01010-23-s0006.jpg]

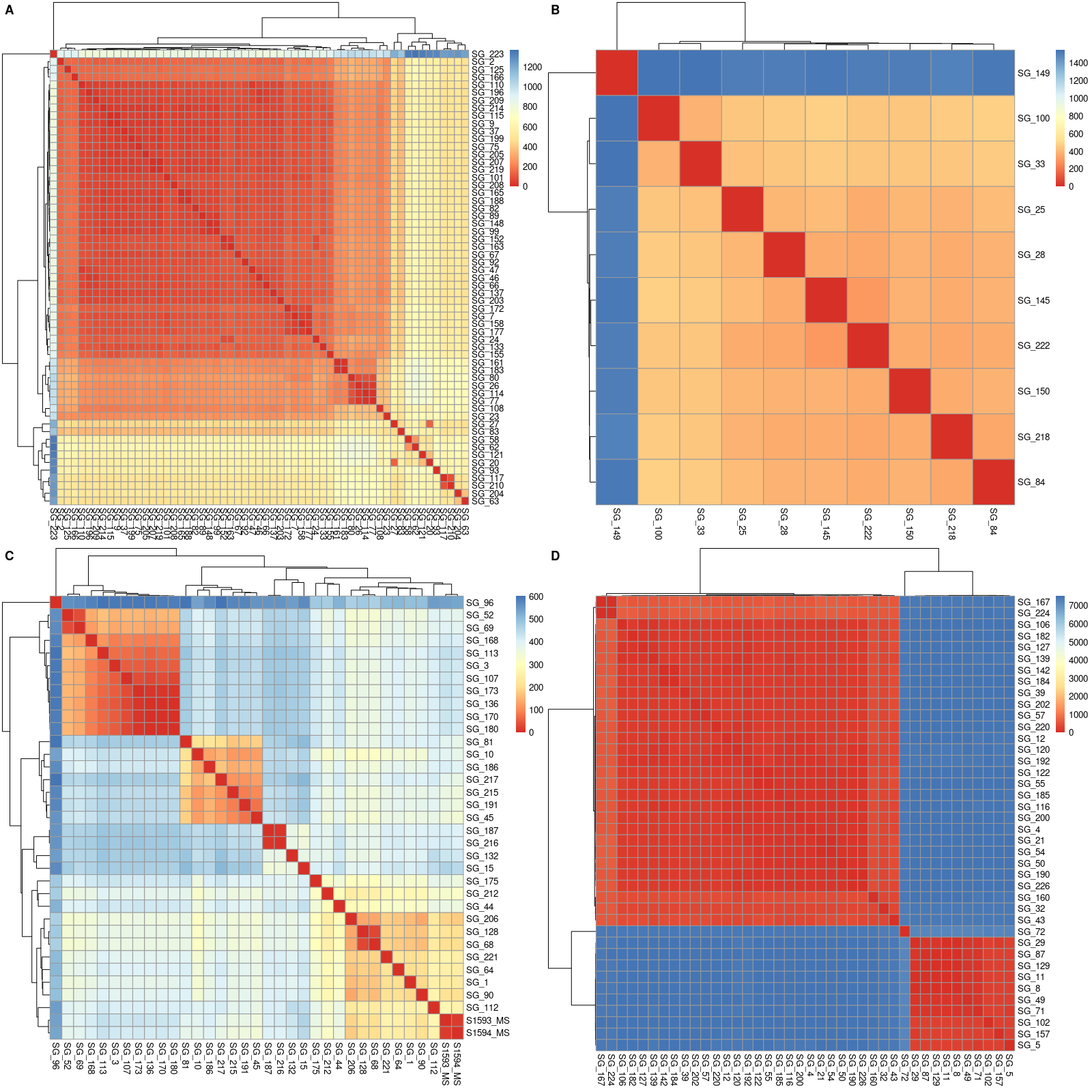

Supplement: Supplemental file 6 — Figure 6. Download spectrum.01010-23-s0007.png, PNG file, 0.3 MB [file spectrum.01010-23-s0007.png]
