# Supplementary material for: Combining Genome Surveillance and Metadata To Characterize the Diversity of Staphylococcus aureus Circulating in an Italian Hospital over a 9-Year Period
Source: Microbiol Spectr. 2023 Jul 17;11(4):e01010-23. doi: 10.1128/spectrum.01010-23 (PMC10433831; doi:10.1128/spectrum.01010-23)
Supplement: Supplemental file 2 — Figure S2. Download spectrum.01010-23-s0003.pdf, PDF file, 0.4 MB [file spectrum.01010-23-s0003.pdf]

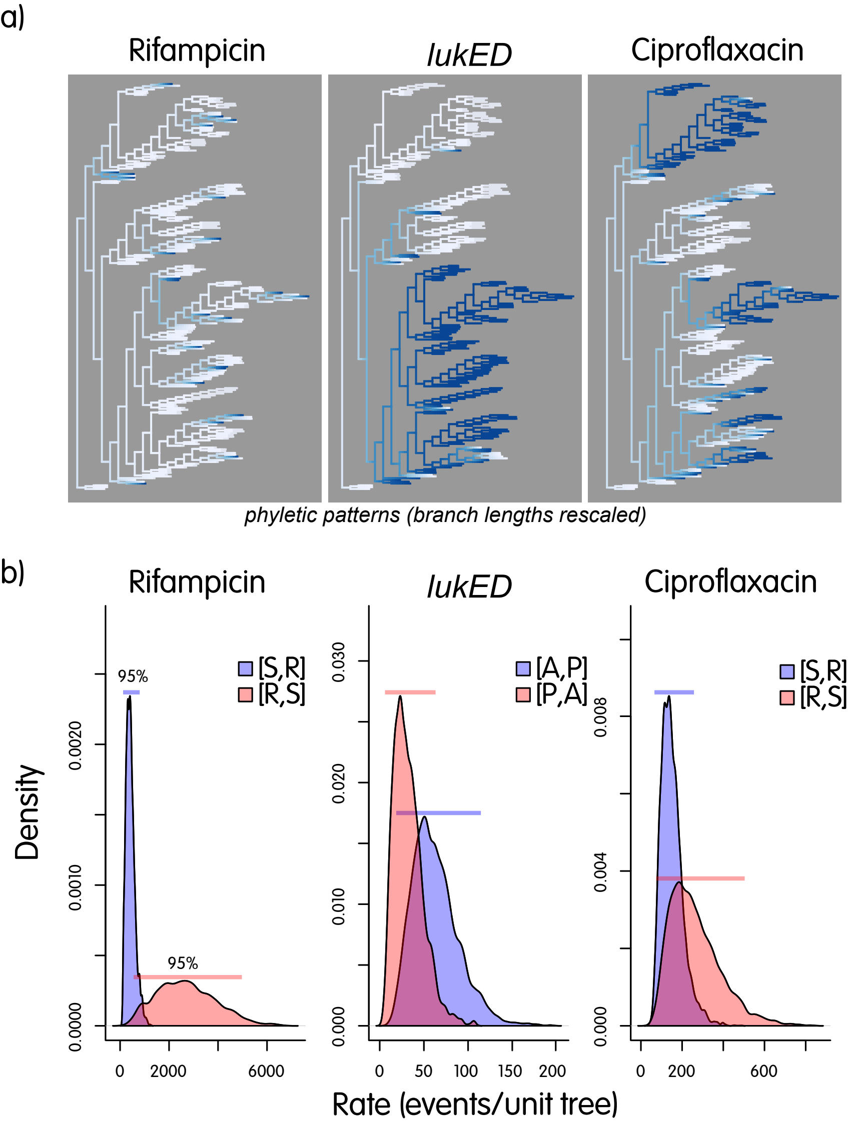

a)
Rifampicin
lukED
Ciproflaxacin
phyletic patterns (branch lengths rescaled)
b)
Rifampicin
lukED
Ciproflaxacin
Density
95%
95%
[S,R]
[R,S]
[A,P]
[P,A]
[S,R]
[R,S]
0.0000
0.0010
0.0020
0.000
0.010
0.020
0.030
0.000
0.004
0.008
0
2000
6000
0
50
100
150
200
0
200
600
Rate (events/unit tree)
