# Supplementary material for: Combining Genome Surveillance and Metadata To Characterize the Diversity of Staphylococcus aureus Circulating in an Italian Hospital over a 9-Year Period
Source: Microbiol Spectr. 2023 Jul 17;11(4):e01010-23. doi: 10.1128/spectrum.01010-23 (PMC10433831; doi:10.1128/spectrum.01010-23)
Supplement: Supplemental file 7 — Supplemental legends. Download spectrum.01010-23-s0008.docx, DOCX file, 0.02 MB [file spectrum.01010-23-s0008.docx]

**SUPPLEMENTARY MATERIAL LEGENDS**

**SUPPLEMENTARY FIGURES**

Supplementary Figure 1: bar plot representations of the dataset by 1a ward stratified on sample types; 1b: antibiotic resistances stratified on sample types; 1c: antibiotic resistance in case of communitary (CA) and hospital (HA) acquired infections; 1d: communitary (CA) and hospital (HA) acquired infections stratified on sample type; 1e: Inpatients and outpatients stratified on sample types.

Supplementary Figure 2: Phylogenetic tree of the entire genomic dataset (n=226). The tree was generated using a core-SNPs alignment with the genome NCTC 8325 (ST36) as reference through the P-DOR pipeline. For each genome, both the isolate name and the ST are displayed. In addition, rare STs (n<3) are coloured in light blue.

Supplementary Figure 3. Frequency of isolates with N antibiotic resistances (x axis) by stratification of the CA and HA isolates also on the basis of methicillin resistance. MS and MR strains are similar in the two CDC categories; MS are devoid of most multi-drug isolates while MR are devoid of strains sensitive to all or most antibiotic tested (anti-symmetric profiles). The cumulative antibiotic resistance profiles (CA and HA) indicate a higher tendency of HA strains to be more multidrug resistant.

Figure 4. Phylogenetic trees of the four main clonal complexes, including genomes obtained in this study (colored) and from the PATRIC database (grey). For clarity, all tip labels are aligned. Local genomes are highlighted by colored circles indicating the sequence type and allowing to detect monophyletic groups exclusively containing genomes from this work, and can be interpreted as representing persistent clones in the hospital.

Supplementary figure 5. Phylogenetic trees of the four main clonal complexes with branch lengths, including genomes obtained in this study (colored) and from the PATRIC database (grey). For clarity, all tip labels are aligned. Local genomes are highlighted by colored circles indicating the sequence type and allowing to detect monophyletic groups exclusively containing genomes from this work, and can be interpreted as representing persistent clones in the hospital.

Supplementary figure 6. heatmaps showing SNP distances between each pair of genomes within the four main clonal complexes. Figures a, b, c and d depict the distances in Clonal complexes 22, 30, 5 and 8 respectively.

**SUPPLEMENTARY TABLES**

Supplementary table 1: Average number of resistances carried by MR and MS strains, showing that MSSA are collectively less commonly multi-drug resistant.

Supplementary Table 2. Time trends of antibiotic resistances. Clindamycin and Erythromycin have opposing time trends in MR (decrease) and MS (increase), as evidenced by the regression coefficient (beta) estimated for time as a predictor of the proportion of resistant isolates.

Supplementary Table 3. Supplementary table 3. resistance profiles of all isolates of the datasets, and of all sequenced isolates grouped through the applied clustering approach. Resistance to each antibiotic is indicated by 1, while 0 means susceptible.

Supplementary table 4: characteristics of the sequenced genomes. isolate identifier; isolation date; genome size; contigs N; Sequence Type; Clonal Complex; Spatype; SCCmec type.

Supplementary Table 5. Total numbers of isolates divided by epidemiological categories Community associated (CA) and hospital associated (HA) and summary of the regression analysis for time trends of MS / MR and CA / HA isolates. Beta refers to the parameter estimated for time as a predictor. A positive coefficient corresponds to an increase in time.

Supplementary Table 6. frequencies of resistance to the different antibiotics stratified by CDC category (HA Hospital acquired, CA community acquired) and differences in the two groups.

Supplementary Table 7: Predictors with a significant association with casualties in a Generalized linear model comprising all patients and the predictors indicated in Materials and Methods. When considering all patients, the most important predictor is Age, together with two predictors associated with the source of infection. While the coefficient for age seems small with respect to all the others, the range of Age is much wider. Additionally, Blood and Respiratory tract are mutually exclusive, and therefore Age is by far the most important predictor of mortality consequent to Staphylococcus aureus infection.

Supplementary Table 8. Phylogenetic signal of virulence factors, antibiotic resistance traits, CDC status and outcome. CC22 is a clonal complex, taken as an example of a “trait” correlated to phylogenetic structure by definition. Values of D less than -1 indicate that a certain trait is in agreement with the topology of the phylogenetic tree, while values of D larger than 1 indicate patterns not following the structure of the tree (overdispersed traits).

Supplementary Table 9. Phylogenetic regression linking genomic properties to the outcome. Only predictors with a p-value less than 0.1 are reported. Negative coefficients correspond to a reduced mortality when the trait is present, viceversa for positive coefficients.

Supplementary table 10. accession numbers of all the BV-BRC genomes used for phylogenetic contextualization.
